# Society Meetings

**Published:** 1908-03

**Authors:** 


### SOCIETY MEETINGS.

---

The Buffalo Academy of Medicine held meetings during January and February as follows:

*Section on Obstetrics and Gynecology.*—Tuesday evening, January 28. Program: Enteroptosis, illustrated by stereopticon slides, J. G. Clark, Professor of Gynecology in the University of Pennsylvania. Discussion led by Matthew D. Mann. Management of pregnancy and some of its disorders, Earl P. Lathrop.

*A Stated Meeting* of the Academy was held Tuesday evening, February 4. The Program of the evening was furnished by the Section on Surgery, as follows: (*a*) The treatment of acute prostatitis and allied complications of gonorrhea, Edward L. Keyes, Jr.. New York; (*b*) Inguinal hernia in females. Operative report of cases, Vertner Kenerson.

*Section on Medicine.*—Tuesday evening, February 11. Program: Proteid Poisons, Victor C. Vaughan, Ann Arbor, Mich.

Owing to the sudden illness of Dr. Vaughan, no meeting was held on Tuesday, February 11.

*Section on Pathology.*—Tuesday evening, February 18. Program: (*a*) Some observations on the sphenoidal sinus, with presentation of specimens, James A. Gibson; (*b*) Demonstration of a new method of determining urinary acidity, A. L. Benedict.

*Section on Obstetrics and Gynecology.*—Tuesday evening, February 25. Program: (*a*) Preparation of patient, Earl P. Lothrop; (*b*) Danger of operating in acute septic pelvic conditions, Sigmund Goldberg.

---

The Medical Society of the State of New York at its 102d annual meeting held at Albany, N. Y., January 27-29, 1908, elected the following named officers for the ensuing year: president, Edward L. Trudeau; vice-presidents, A. G. Root, John Wheeler, and M. C. Hawley; secretary, Wisner R. Townsend; treasurer, Alexander Lambert; chairman of the Scientific Committee, George H. Neuman; chairman of the Committee on Public Health, J. L. Heffron; chairman of the Committee on Legislation, F. Van Fleet; chairman of the Committee of Arrangements, W. J. Nellis; delegates to the American Medical Association, for one year, R. F. Weir, and Charles Jewett; for two years, W. R.

Townsend, D. C. Moriarta, E. B. Angell, J. C. Bierwith, and Albert Vander Veer; alternate delegates for two years, Drs. Thornton, Brown, Little, Glass, Dunning, and Stover; alternate delegates for one year, Drs. J. A. Fordyce, A. H. Terry, W. T. Mulligan, C. G. Rossman, and Burrols. Mr. J. T. Lewis was re-appointed the attorney for the society.

Dr. E. L. Trudeau subsequently declined the office of president and according to the by-laws Dr. A. G. Root, of Albany, will assume the office for the ensuing year.

---

The Association of American Medical Colleges will hold its next annual meeting at the Hotel Euclid, Cleveland, March 16, and 17, 1908, under the presidency of Dr. H. B. Ward, of Lincoln, Neb. All those interested in medical education are cordially invited to attend and participate in the discussions. It is desired that the attendance include not only medical educators, but educators in general, members of state medical examining boards, and representatives of allied associations which discuss educational problems.

---

The Rochester Academy of Medicine held meetings during February as follows:

*Section on Obstetrics, Gynecology, and Pediatrics.*—Wednesday evening, February 5. Program: The clinical significance of glycosuria during pregnancy, J. Whitridge Williams, Professor of Obstetrics, Johns Hopkins University, Baltimore.

*Section on Surgery.*—Wednesday evening, February 12. Program: A cause of pain in the upper back, C. Wentworth Hoyt (by invitation); Diseaes of the hip occurring in adults, Ralph R. Fitch.

*Section on General Medicine.*—Wednesday evening, February 19. Program: Human asymmetry, William S. Ely; Acute anterior poliomyelitis, Robert G. Cook; Demonstration of the action of the valves of the heart, John W. Radu (by invitation).

*Section on Public Health, etc.*—Wednesday evening, February 26. Program: The systematic examination and treatment of children; a first principle in maintaining a high standard of public health and efficiency. A symposium: Dr. Snell, Ocular defects; Dr. McDowell, Ear, nose and throat defects; Dr. Rosenthal, General functional and physical defects; Dr. Goler, School inspection; its accomplishments, and suggestions for its progress.

The New York Medico-Legal Society and The American International Congress for Tuberculosis will meet in joint annual session at Chicago, June 1, 2, and 3, 1908. The local committee of arrangements consists of Denslow Lewis and Thomas Bassett Keyes of Chicago and E. S. McKee, of Cincinnati. The latter was appointed to take the place of Nicholas Senn, recently deceased. The permanent offices of both societies are at 39 Broadway, New York.

The Homeopathic Medical Society of the State of New York at its annual meeting held at Albany, February 11 and 12, 1908, elected the following-named officers for the ensuing year: president, William S. Garnsey, Gloversville; vice presidents, Frank B. Seitz, Buffalo; William H. Nickelson, Adams; Robert C. Woodman, Middletown; secretary, Bert B. Clark, New York; treasurer, Reeve B. Howland, Elmira; necrologist, John L. Moffat, Brooklyn.

